# SilhouetteScoreinR: Beyond traditional network layouts by leveraging local cohesion and nearest neighbor separation

**DOI:** 10.1016/j.mex.2025.103622

**Published:** 2025-09-11

**Authors:** Hua-Ying Chuang, Willy Chou

**Affiliations:** a Department of Internal Medicine, Chi Mei Medical Center, Chiali District, Tainan, Taiwan; b Institute of Physical Education, Health and Leisure Studies, National Cheng Kung University, Tainan, Taiwan; c Department of Nursing, Chung Hwa University of Medical Technology, Tainan, Taiwan; d Department of Physical Medicine and Rehabilitation, Chi-Mei Medical Center, Liuying Chimei Hospital, Tainan, Taiwan; e Department of Leisure and Sports Management, CTBC University, Tainan, Taiwan

**Keywords:** Silhouette score, Explicit and implicit layouts, Bibliometric analysis, Cluster Validation, Network Community Detection

## Abstract

The silhouette score (SS) quantifies how well each entity fits its assigned cluster by contrasting within‐cluster cohesion with nearest other–cluster separation. Although common in other fields, SS is rarely used in bibliometrics. Using 2,252 *MethodsX* articles (2020–2024), we show how SS evaluates clustering quality in co-word networks and author collaborations, independent of the chosen algorithm. We provide R scripts to compute SS for explicit (geographic/known coordinates) and implicit (PCA/UMAP) layouts and introduce a two-axis visualization that plots publication count against SS. The framework highlights coherent clusters (high SS) and flags boundary or misassigned entities (low/negative SS) that standard network plots can obscure. This improves interpretability at term, cluster, and corpus levels and supports more defensible decisions about labels, membership, and follow-up analysis. Code is released for replication and reuse; sensitivity to distance metrics and data regimes is discussed to guide application across bibliometrics and related domains.

• Silhouette Scores Reveal Outliers: Silhouette scores not only validate cluster cohesion but also uncover meaningful outliers—insights often missed in traditional network layouts.
• Novel Visualization Approach: Combining silhouette scores with publication counts enables a more nuanced visualization of co-word and collaboration networks.
• Applied to Bibliometrics: This study applies silhouette analysis to 2252 MethodsX articles, offering new tools for evaluating clustering quality in bibliometric research.

## Graphical abstract

**Figure:**
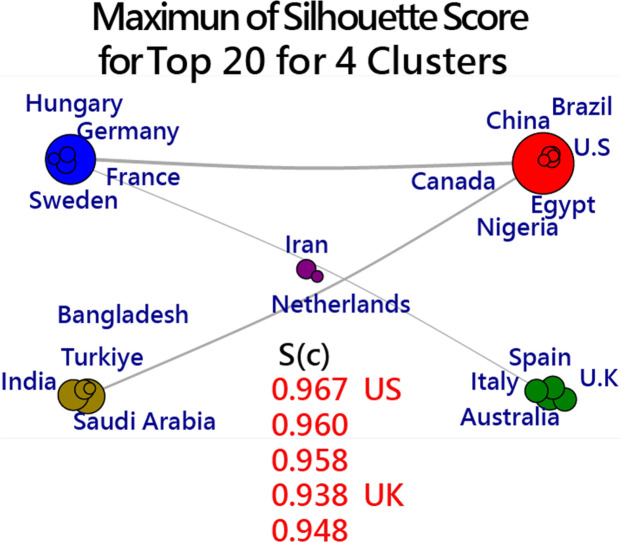

## Related research article

*Rousseeuw PJ. Silhouettes: a graphical aid to the interpretation and validation of cluster analysis. J Comput Appl Math. 1987;20:53–65. doi:10.1016/0377–0427(87)90,125–7*

## Specifications table

**Table:** 

**Subject area**	Mathematics and Statistics Economics, Econometrics and Finance Social Sciences Sciences Medical Sciences
**More specific subject area**	Visual representation
**Name of your method**	**Silhouette Score computed in R** script [**SilhouetteScoreinR**]
**Name and reference of original method**	[9] Rousseeuw PJ. Silhouettes: a graphical aid to the interpretation and validation of cluster analysis. J Comput Appl Math. 1987;20:53–65. doi:10.1016/0377–0427(87)90,125–7
**Resource availability**	**SilhouetteScoreinR** at https://raschonline.com/raschonline/cbp.asp?cbp=SilhouetteScorefromcountry2 https://raschonline.com/raschonline/cbp.asp?cbp= SilhouetteScores https://github.com/smilechien/novel-silhouette-plots/

## Background

Clustering is an unsupervised technique that partitions data into distinct groups, or clusters, so that items within the same cluster are similar while those in different clusters are dissimilar [1]. Its goal is to uncover natural structures in data [[2], [3], [4], [5]], with applications in psychology [6], biology [5], pattern recognition [4], image processing [7], and computer security [8].

Once clustering is performed, an important question arises: How well does the partition fit the data? This is critical for two reasons [1]. First, there is no universally optimal algorithm—different methods or settings can produce different results. Therefore, multiple candidate partitions often need to be generated and compared. Second, many algorithms require specifying the number of clusters (the *k* parameter [2]) in advance, even though this is rarely known beforehand. Practitioners typically test several *k* values and evaluate the results—a process known as cluster validation [2].

One common validation measure is the silhouette score (SS) [9], which quantifies clustering quality. There are two types of layouts for evaluating SS: (1) *explicit layouts*, which use measured distances, and (2) *implicit layouts*, which rely on latent or inferred distances. SS is widely used in bibliometric analyses (e.g., with CiteSpace [10]) to assess cluster validity [[11], [12], [13], [14], [15]]. However, comprehensive explanations and reproducible R scripts [16] are rarely provided, limiting the ability of future researchers to replicate these analyses.

Traditional network plots (e.g., 2D plots without count and SS on axes) often fail to capture the full richness of cluster insights. In this study, we provide R scripts illustrating the use of three data types—time-series rectangular data, co-word occurrence data, and survey feature-variable data—as examples of both explicit and implicit layouts.

## Method details

### Data source

We retrieved metadata for 2252 articles published in the Journal of *METHODSX f*rom 2020 to 2024, as of January 22, 2025, using the Web of Science Core Collection (WoSCC) database. Study data and guidance to this research are deposed in Appendices 1 and 2.

Since all articles stored in WoSCC do not contain identifiable patient information, ethical review approval was not required.

### Silhouette score (SS)

The silhouette score quantifies how well a data point fits within its assigned cluster compared to other clusters. Ranging from –1 to +1, higher values indicate greater cohesion within clusters and clearer separation between them. It provides an intuitive, single-number diagnostic for assessing clustering quality—helping researchers move beyond guesswork to rigorously validate their results.

#### How to compute the SS

Assume the data have been clustered via any technique, such as k-medoids or k-means, into k clusters. For data point i$\in C_{I}\;$(data point i in the cluster$C_{I}$),

(1) $$a\left(i\right)=\frac{1}{\left|C_{I}\right|-1}\sum_{j\in C_{I},j\neq i}d\left(i,j\right),$$

Let a(i) be defined as the mean distance between point i and all other data points in the same cluster. Here, ∣$C_{I}$∣ denotes the number of points in cluster $C_{I}$, and d(i,j) represents the distance between points i and j within that cluster. The formula divides by ∣$C_{I}$∣−1| because the sum excludes the self-distance d(i,i). Intuitively, a(i) measures how well point i is assigned to its cluster—the smaller the value of a(i), the better the assignment.

We define the mean dissimilarity of point i to another cluster $C_{J}$​ (where $C_{J}$≠CI) as the average distance from i to all points in $C_{J}$​:

(2) $$\text{mean}\;\text{dissimilarity}=\frac{1}{\left|C_{J}\right|}\sum_{j\in C_{J}}d\left(i,j\right),$$

For each data point i∈$C_{I}$​, we then define:

(3) $$b\left(i\right)=\min_{J\neq I}\frac{1}{\left|C_{I}\right|-1}\sum_{j\in C_{I},j\neq i}d\left(i,j\right),$$

Here, b(i) represents the smallest (as indicated by the min operator) mean distance from i to any other cluster—that is, any cluster to which i does not belong. The cluster that achieves this minimum value is called the neighboring cluster of i, since it represents the next best fit for that point outside of its own cluster.

We then define the silhouette value s(i) for point i as:

(4) $$s\left(i\right)=\left\{ \begin{array}{c} \frac{b\left(i\right)-a\left(i\right)}{maxa\left(i\right),b\left(i\right)},\;if\;|CI|>1\\ 0,\;if\;|CI|=1\; \end{array}\right.,$$

Where a special case with one member in a cluster, while ∣CI∣=1 makes s(i)=0 in the bottom of formula (4).

By definition, the silhouette value satisfies:

(5) −1≤s(i)≤1,

It is important to note that a(i) is not defined for clusters of size 1. In such cases, we conventionally set s(i)=0. This choice is arbitrary but neutral, as it lies at the midpoint of the possible silhouette range, from −1 to 1.

#### Examples for the computation of SS

For instance, there are 3 clusters, including A, B, and C, with 2 data points, respectively:

Cluster A: P1 = (0, 0) and P2 = (0, 2)
Cluster B: P3 = (10, 0) and P4 = (12, 0)
Cluster C: P5 = (5, 10) and P6 = (5, 12)

We are going to compute a(i), b(i), and s(i) specifically for P1. Readers can do it for other points if it is interesting.

**Step 1: Distances within Cluster A (P1’s cluster):**

Cluster A members: P1, P2

Distance P1 ↔ P2:

$$d\left(P1,P2\right)=\surd {\left(0-0\right)}^{2}+{\left(0-2\right)}^{2}=2$$

There are only 2 points in A → P1′s only other neighbor is P2. a(1) = average distance to same cluster: a(1)=2

The result reveals that a(1) = 2

**Step 2: Distances to other clusters**

Now we need to find b(1) = average distance to the nearest other cluster.

We do this cluster by cluster.

To Cluster B (P3, P4):

P1 to P3 and P4:

$$d\left(P1,P3\right)=\surd {\left(0-10\right)}^{2}+{\left(0-0\right)}^{2}=10$$

$$d\left(P1,P4\right)=\surd {\left(0-12\right)}^{2}+{\left(0-0\right)}^{2}=12$$

Average distance to Cluster B is (10+12)/2 = 11

The result reveals that b(1) for *B* = 11

Next, to Cluster C (P5, P6)

$$d\left(P1,P5\right)=\surd {\left(0-5\right)}^{2}+{\left(0-10\right)}^{2}=\surd 125\approx 11.18$$

$$d\left(P1,P6\right)=\surd {\left(0-5\right)}^{2}+{\left(0-12\right)}^{2}=\surd 169=13$$

Average distance to Cluster C is (11.18+13)/2≈12.09

The result reveals that b(1) for C ≈ 12.09

We then pick the closest cluster as b(1) = min(b_B, b_C) = min(11, 12.09) = 11

The result for b(1) is 11

**Step 3: Compute s** (1)

The silhouette score:

$$s\left(1\right)=\frac{b\left(i\right)-a\left(i\right)}{maxa\left(i\right),b\left(i\right)}=\frac{11-2}{11}=\frac{9}{11}\approx 0.818$$

So, s(1) ≈ 0.818

**Step 4: Final Answer for P1**

• a(1) = 2
• b(1) = 11
• s(1) ≈ 0.818

We summary Table 1 for P1 as below:

**Table 1:** Summary for individual Point 1(P1).

Metric	Value	Meaning
a(1)	2	Avg distance to own cluster (tightness)
b(1)	11	Avg distance to closest other cluster
s(1)	0.818	Silhouette score = separation vs. tightness

The cluster S(c) can be obtained by the mean of s(i) in each cluster c. The overall S(o) is computed by the mean of S(c) across all clusters.

#### Examples of explicit layouts for ss characteristics

**Fig. 1:**
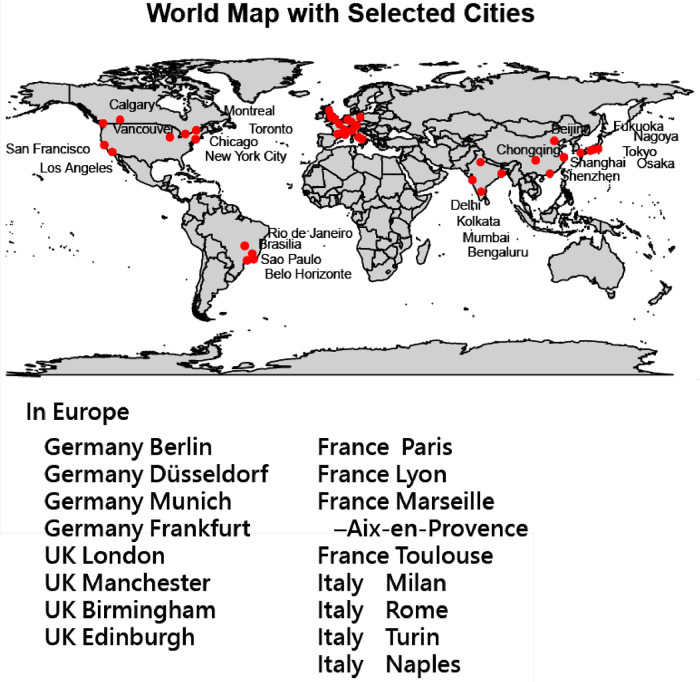
Silhouette scores for the top 4 cities in the top 10 GDP countries.

One example of explicit layout (i.e., distances are consistent with a network layout) regarding *silhouette scores* in world-map context, especially *by distance between top 4 cities in the Top 10 GDP countries*. Fig. 1 [17] *displays Silhouette scores here reflect how clearly a country’s top cities cluster together* versus *differ from cities in other countries, based on their geographic distances.*

We can see that map-based explicit layout illustrating how geographic distance between major cities affects clustering quality. Higher silhouette scores (e.g., Brazil 0.93, Japan 0.77) indicate tightly clustered, well-separated city groups. Moderate scores (e.g., UK 0.68, India 0.62) suggest more spread within clusters, while low or negative scores (e.g., US –0.10, Canada 0.00) reveal dispersed, poorly defined clusters.

**Fig. 2:**
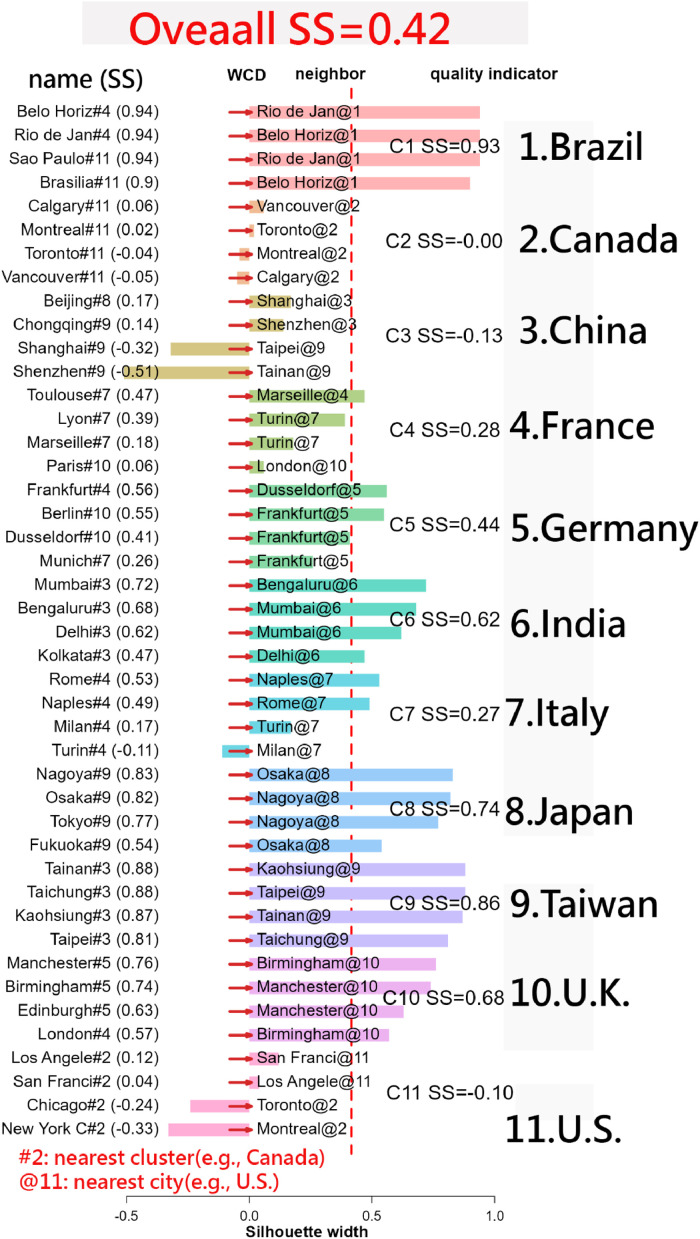
Silhouette plot for the top 4 cities in the top 10 GDP countries and Taiwan.

Key messages can be conveyed here in Fig. 2 [18]. Higher silhouette scores signal geographically compact, distinct city groups within a country(e.g., Brazil and Taiwan); lower or negative scores mean cities are geographically dispersed, weakening their cluster identity(e.g., China, Canada, and the U.S.).

### R package used in this study

Visualizations in R created on the R platform [19,20] were used to compute the SS for each cluster and draw networks with count and SS on y-/x- axes, respectively. All data processing and statistical analyses were conducted using R software version 4.1.3 [16], and the generation of time-series visualizations and result output was performed within the RStudio integrated development environment [21].

### Relation to other validation indices

Silhouette score [9] evaluates each term’s cohesion vs separation using the term’s average intra-cluster distance a(i) and nearest-other-cluster distance b(i), yielding interpretable, pointwise diagnostics and per-cluster/overall means.

In graph settings derived from co-occurrence networks, modularity Q [22,23] measures how many edges fall within communities relative to a null model; it can favor partitions with dense inter-cluster bridges or suffer resolution limits [24] that merge small, well-separated groups—cases where Q may be high while SS is low because many terms lie nearer to another cluster than to their own.

Centrality (e.g., degree, betweenness, eigenvector) characterizes node role (hubs, brokers) rather than cluster fit; boundary hubs often have high centrality but low SS [25,26].

Traditional partition indices such as Calinski–Harabasz (CH) [27] and Davies–Bouldin (DB) [28] provide global summaries: CH rewards high between-/within-cluster dispersion ratios (tends to prefer compact, spherical clusters), while DB penalizes high within-cluster scatter relative to centroid separations (lower is better).

In practice, use SS when we need term-level and cluster-level interpretability and to flag outliers; use CH/DB to help choose k under vector-space models; and use Q to assess community structure on networks, reporting divergences between Q and SS as evidence about density vs distance structure.

## Method validation

### Implicit layouts by co-word occurrence data

Implicit layouts depict latent distances(e.g., with CiteSpace [10]) to produce network charts [[11], [12], [13], [14], [15]] through co-word occurrences in bibliometric research) rather than true network topology, leading to visual distances that do not match direct connections.

**Fig. 3:**
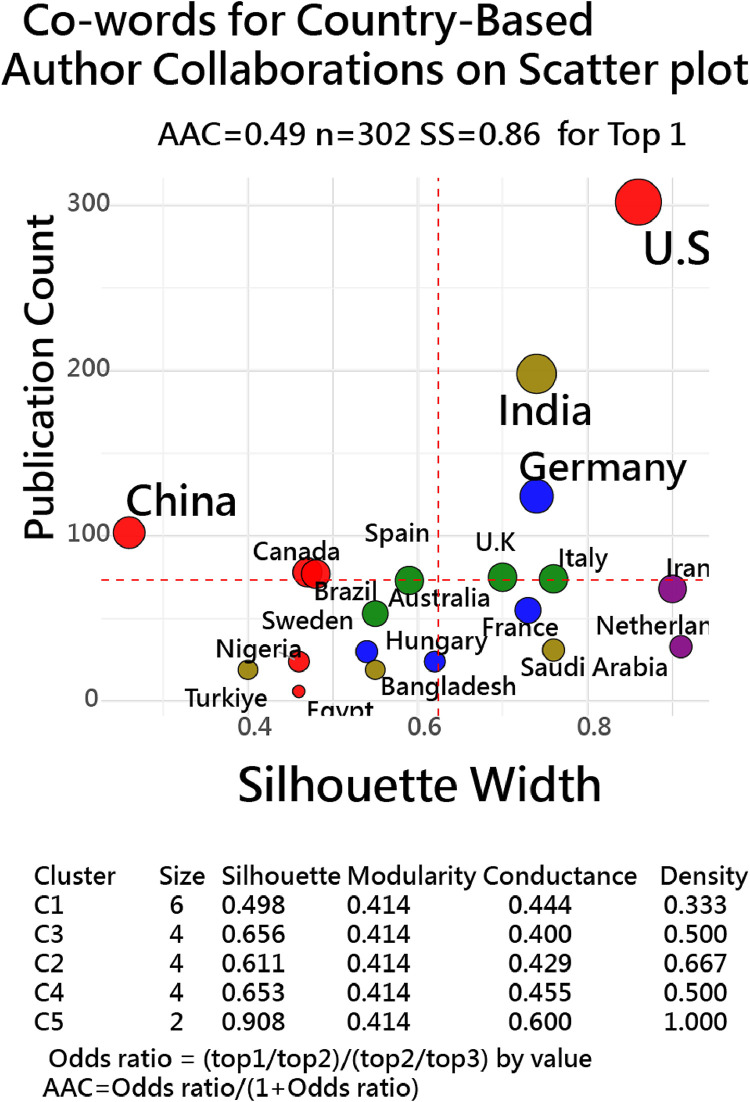
Country-based collaboration network plot using FLCA clustering [33,34] with co-word data(indicating that **the US has the highter SS and publication count in top 20 countries: SS≈0.6–0.8** implies tight, separated clusters; **SS≈0** ambiguous; **SS<0** likely misfit/outlier).

Fig. 3 [19] shows two-dimensional network visualization with coword occurrences of country-based author collaborations showing publication count on the y-axis and silhouette score on the x-axis. Five distinct clusters are observed, revealing more detailed silhouette score distribution than traditional node-grouping charts [[11], [12], [13], [14], [15]]. This approach helps identify cluster separation and cohesion among countries in co-word collaborations.

Importantly, outliers-such as the U.S.-can be identified based on the silhouette score (SS) distance from others, reflecting publication volume and collaboration levels that far exceed those of peers within the same cluster. Notably, such distinctions are not evident in traditional network layouts.

### Implicit layouts by time-series data

**Fig. 4:**
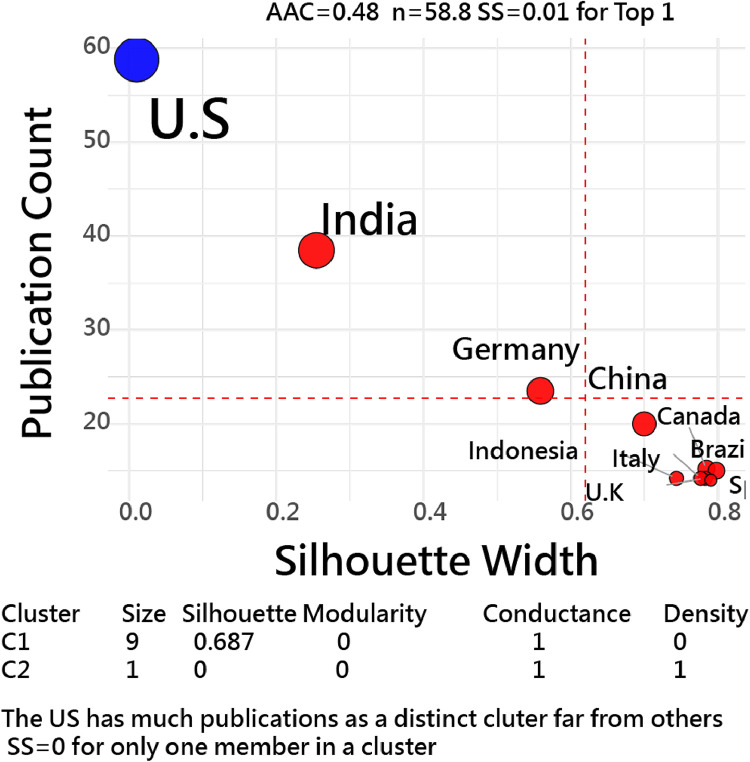
Time-series data for top 10 countries using FLCA clustering [33,34](indicating that **the US has SS at zero due to one term in the cluster: SS≈0.6–0.8** implies tight, separated clusters; **SS≈0** ambiguous; **SS<0** likely misfit/outlier).

Fig. 4 [20] presents a two-dimensional network plot of the top 10 countries by publication counts over the years using time-series data, generated using an R script [20]. This approach reveals two distinct clusters led by the U.S. and India, respectively, with lower silhouette scores (e.g., zero for the U.S. due to a single node in the cluster, 0.25 for India outside others in the same cluster).

### Implicit layouts by survey data

The Zoo dataset [29] from the UCI Machine Learning Repository contains 101 animals described by 16 categorical features (e.g., hair, feathers, eggs, milk, airborne, aquatic, predator) and a class label indicating one of seven animal types (e.g., mammal, bird, reptile). Designed for classification tasks, it supports analysis of how well machine learning models can distinguish species based on simple traits with silhouette scores.

**Fig. 5:**
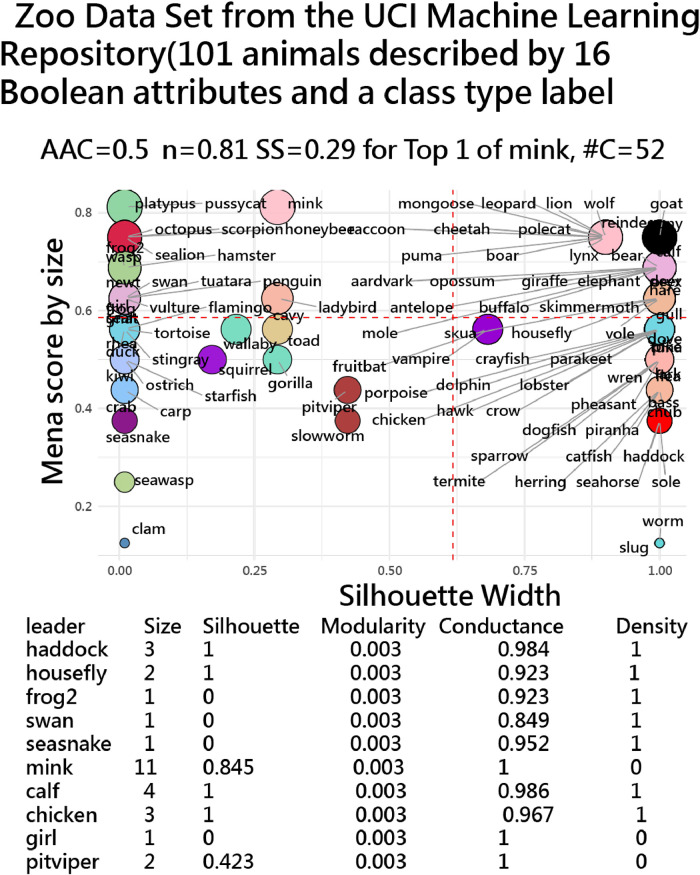
Clustering of featured variables using Euclidean distance algorithm [20] (indicating that **52 clusters of animals by size in color with SS≈0.0–1.0** implies weak and tight, separated clusters; **SS≈0** ambiguous; **SS<0** likely misfit/outlier).

Fig. 5 shows clustering of top 20 animals from the Zoo dataset into 52 groups based on the classification in data [29], with two distinct groups of silhouette scores measuring how well each animal fits within its assigned cluster using Euclidean distance algorithm with R script [20].

**In Top panel, a** 2D plot positions animals by count (y-axis) and silhouette score (x-axis). We found that (1) High silhouette width (>0.7): strongest, well-defined groups (big mammals & some predators); (2) Low silhouette width (<0.3): animals with fuzzy identities (clam, seawasp, worm, slug); (3) Cluster colors: work like “animal families” — mammals, sea creatures, reptiles/insects, etc.; and (4) Bubble size: larger animals (or more dominant species in dataset) stand out as Leaders within clusters.

**Fig. 6:**
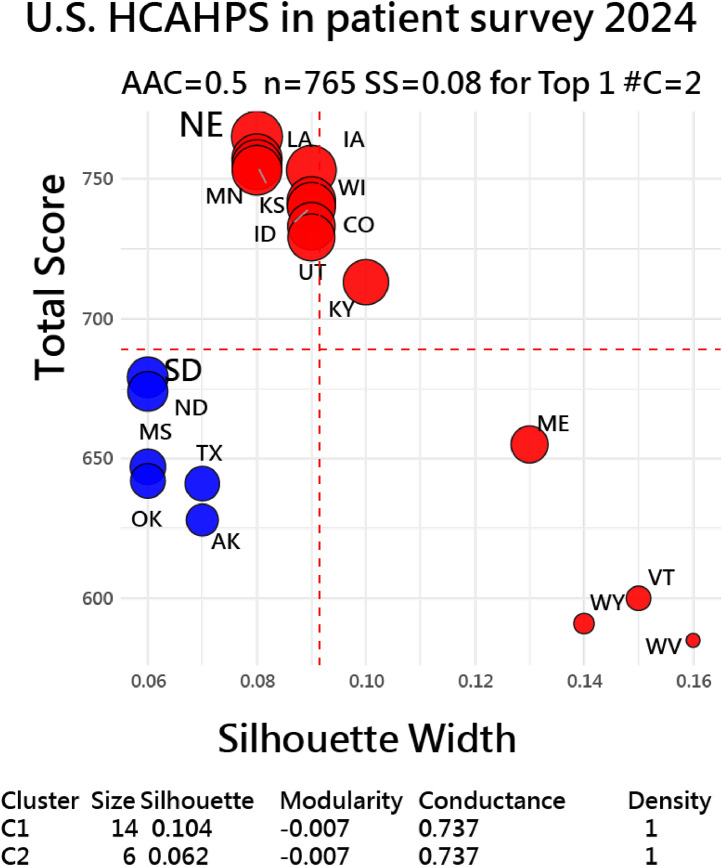
U.S. HCAHPS survey results [30,31] via R script [32] in two distinct clusters for top 20 US states,with weak and moderate silhouette scores.

Another example in Fig. 6 was from the U.S. HCAHPS survey results [30,31] via R script [32]. Two clusters were observed in top 20 US states, with moderate and weak silhouette scores, respectively.

### Applying silhouette analysis across domains: distances, layouts, and interpretation

• Bioinformatics: Compute SS on cosine/correlation after variance-stabilizing normalization (e.g., log-CPM/TPM with batch correction). Use PCA/UMAP only for display. SS ≈0.5–0.7+ signals coherent programs; ∼0 suggests transitional/heterogeneous states; <0 indicates misassignment/batch. Report cluster-mean SS with marker enrichment.
• Customer segmentation: For mixed data, use Gower distance (optionally weighted) with medoid-based clustering. Visualize via PCoA/MDS. High SS implies distinct, homogeneous segments; low SS implies overlap or excessive k. Assess stability by bootstrap; report the distribution of segment-level SS.
• Anomaly/fraud analytics: Treat SS as an outlier signal with context-specific local distances (scaled Euclidean/Manhattan, class-conditional Mahalanobis, k-NN graph). Keep layouts diagnostic. Very low/negative SS flags near-other-cluster or isolated cases; set thresholds with temporal windows and cost-sensitive precision/recall.
• Transportation/urban studies: Use explicit geographic layouts; compute SS on Haversine or network path distances. High SS marks compact zones; low SS indicates corridors, polycentric areas, or connectivity-driven clusters. Pair SS with shape/elongation metrics to separate dispersion from linear geography.
• Interpretation across domains: Mean SS >0.70 = strong; 0.50–0.70 = acceptable; 0.25–0.50 = weak; <0 = likely misassignment. Combine term/cluster SS with CH/DB (vector spaces) or modularity Q (graphs) to disentangle distance- vs density-driven structure.
• Sports for players’ performance: an example of Women's 10 m platform final in Paris 2024 [35].

### To tests the study hypothesis

Many clustering algorithms require the number of clusters (often referred to as the *k* parameter [2]) to be specified in advance, besides the FLCA [31,33,34]. As a result, numerous indicators are used to evaluate clustering quality [1]. The silhouette score is one such measure [9]. Quality indicators such as modularity and centrality have been also considered to assess network quality [12].

While higher silhouette scores typically indicate stronger cohesion within a cluster, lower silhouette scores should not be overlooked. They can highlight meaningful outliers—such as the U.S. in Fig. 2, Fig. 3—which, despite belonging to a cluster, exhibit distinct separation from other members. This distinction is often invisible in traditional network layouts but becomes evident through silhouette-based visualization (Table 2).

**Table 2:** Summary of R scrips used in this study with SilhouetteScoreinR.

Item	Visualization Method	R script	Figure	Note
1	World map data	[17,18]	1,2	Distances derived by coordinates
2	Coword occurred data	[19]	3	Replace data in country.csv
3	Time-series data	[20]	4	Paste rectangular data into R
4	Featured variables	[20,29]	5	Using zoo_data.csv
5	Survey & performance	[21]	6	Basket model used for testdata [30,31]
6	Data and R scripts	[37]	1–6	https://github.com/smilechien/novel-silhouette-plots/

## Limitations

Despite the strengths of SilhouetteScoreinR applied to social science, the study has several limitations, listed below:

1. Many clustering algorithms (excluding FLCA [31,33,34]) require the number of clusters (the k parameter) to be specified in advance. This can lead to variability in results depending on the choice of k.
2. Cluster validation depends on distance measures and layout assumptions (explicit or implicit). Different layouts (e.g., force-directed vs. coordinate-based) may emphasize or obscure cluster separation in different ways.
3. The SS measures cohesion and separation but depends on the chosen distance metric. Its interpretation can vary by data type (e.g., geographic, co-word, time series, and survey data) and may fail to capture complex network relationships if not carefully considered in advance by researchers.
4. Traditional network plots that do not use count and SS on axes may fail to convey the full richness of cluster-related insights, potentially oversimplifying the underlying structure.
  Although R scripts are provided for three data types (time-series, co-word occurrence, and survey features), their generalizability to other contexts remains untested.
5. The study introduced only the Euclidean distance method, with no discussion of a correlation-based approach (e.g., the R script provided in Reference [36]). To assess sensitivity by repeating the analysis with 2–3 distance metrics (e.g., Euclidean, cosine, Gower) with R scripts is helpful in the future, with reporting the change in average silhouette (Δs̄). Choose k by inspecting the s̄(k) curve and cross-checking Calinski–Harabasz and Davies–Bouldin; for networks, also examine modularity Q. Evaluate stability with bootstrap resampling and summarize per-cluster mean-SS distributions, flagging wide IQRs as unstable. When indices disagree, favor the distance that matches data semantics (e.g., cosine for text, Gower for mixed data) and verify robustness via small perturbations (subsampling, feature noise, random seeds).

This study demonstrates that silhouette scores offer a powerful supplement to traditional network layouts by revealing both cohesive clusters and meaningful outliers. Applied to MethodsX articles, this approach enhances the evaluation of co-word and author collaboration structures, offering a more nuanced and informative perspective for bibliometric analysis.

## Ethics statements

Our study did not involve human subjects, animal experiments, or data collected from social media platforms. The work presented is based on the development and application of computational algorithms for bibliometric visualization using publicly available datasets from Web of Science. All data used were obtained from openly accessible sources, and no personally identifiable or sensitive information was involved. The methodologies applied comply with ethical research standards and adhere to the principles of transparency, reproducibility, and responsible data handling.

## CRediT author statement

HYC: Conceptualization, Methodology, Formal analysis, Writing – original draft.

WC: Methodology, Data curation, Investigation, Writing – review & editing.

LC: Conceptualization, Supervision, Validation, Data interpretation, Writing – review & editing.

## Declaration of competing interest

The authors declare that they have no known competing financial interests or personal relationships that could have appeared to influence the work reported in this paper.

## Data Availability

The data are public and available in Appendices: Appendix 1: XLS(study data), Appendix 2: PDF(How to conduct this study)

## Appendix Supplementary materials

Image, application 1
