# Supplementary material for: SilhouetteScoreinR: Beyond traditional network layouts by leveraging local cohesion and nearest neighbor separation
Source: MethodsX. 2025 Sep 11;15:103622. doi: 10.1016/j.mex.2025.103622 (PMC12466245; doi:10.1016/j.mex.2025.103622)

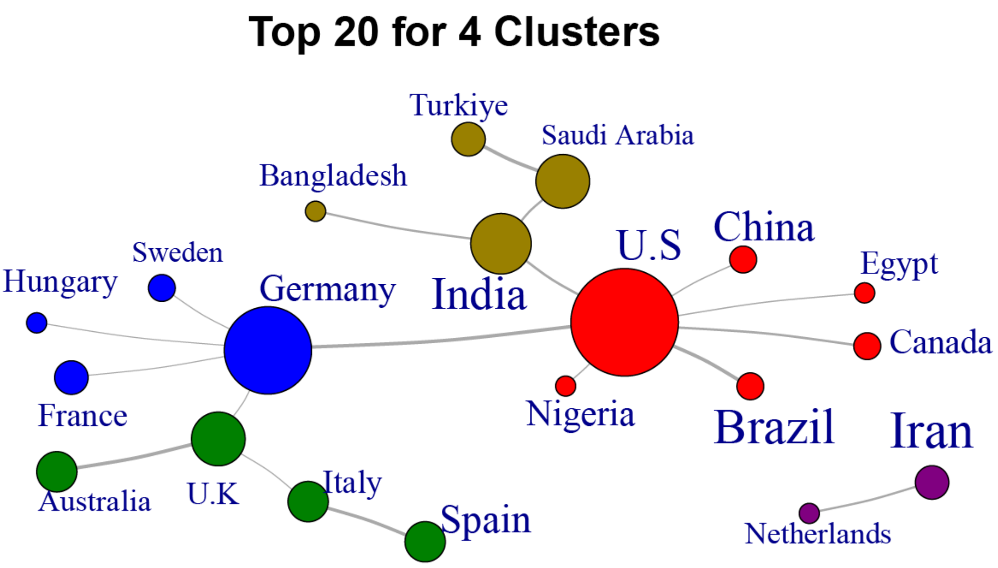

Figure 1 Traditional Network for Country-based author collaborations

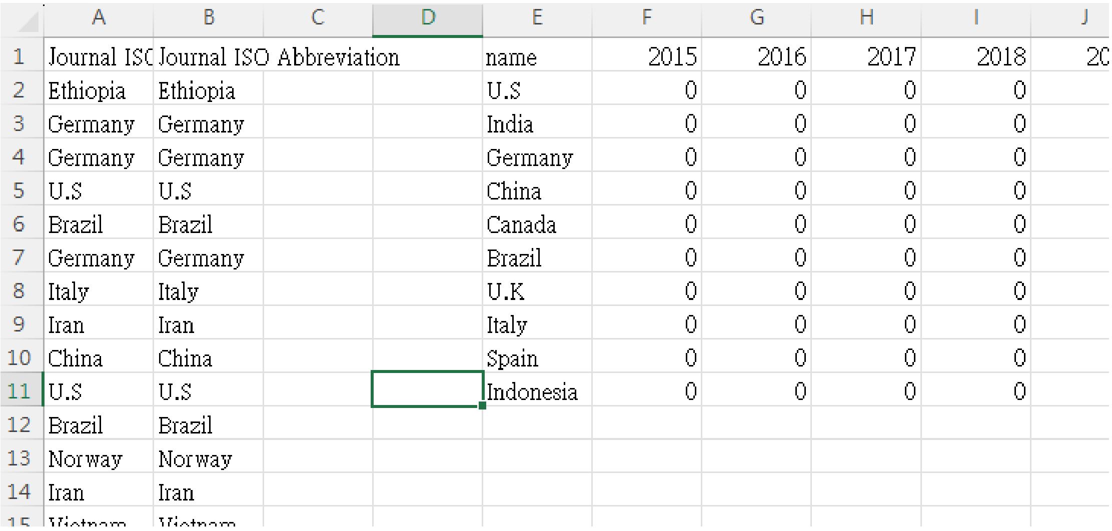

	A	B	C	D	E	F	G	H	I	J
1	Journal ISC	Journal ISO Abbreviation			name	2015	2016	2017	2018	20
2	Ethiopia	Ethiopia			U.S	0	0	0	0	
3	Germany	Germany			India	0	0	0	0	
4	Germany	Germany			Germany	0	0	0	0	
5	U.S	U.S			China	0	0	0	0	
6	Brazil	Brazil			Canada	0	0	0	0	
7	Germany	Germany			Brazil	0	0	0	0	
8	Italy	Italy			U.K	0	0	0	0	
9	Iran	Iran			Italy	0	0	0	0	
10	China	China			Spain	0	0	0	0	
11	U.S	U.S			Indonesia	0	0	0	0	
12	Brazil	Brazil								
13	Norway	Norway								
14	Iran	Iran								
15	Vietnam	Vietnam								

Figure 2 Coword data and time-series data

	A	B	C
1	Journal ISO	Journal ISO	Abbrevi
2	Ethiopia	Ethiopia	
3	Germany	Germany	
4	Germany	Germany	
5	U.S	U.S	
6	Brazil	Brazil	
7	Germany	Germany	
8	Italy	Italy	
9	Iran	Iran	
10	China	China	
11	U.S	U.S	
12	Brazil	Brazil	
13	Norway	Norway	
14	Iran	Iran	
15	Vietnam	Vietnam	

F

Figure 3 saved as country.csv

https://raschonline.com/raschonline/cbp.asp?cbp=SilhouetteScorefromcountry2

Zoo Data Set from the UCI Machine Learning Repository(101 animals described by 16 Boolean attributes and a class type label

AAC=0.5 n=0.81 SS=0.29 for Top 1 of mink, #C=52

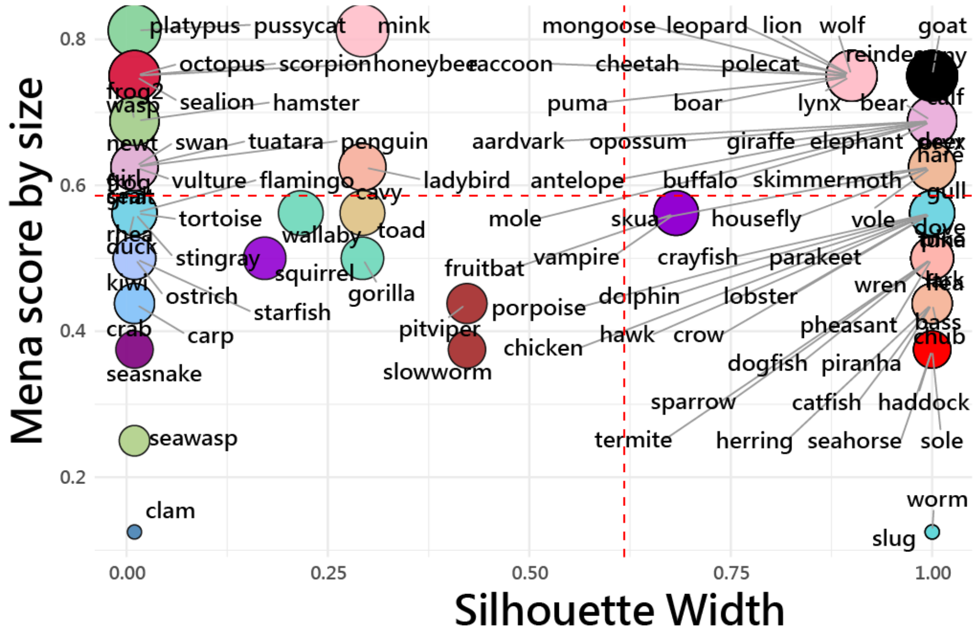

leader	Size	Silhouette	Modularity	Conductance	Density
haddock	3	1	0.003	0.984	1
housefly	2	1	0.003	0.923	1
frog2	1	0	0.003	0.923	1
swan	1	0	0.003	0.849	1
seasnake	1	0	0.003	0.952	1
mink	11	0.845	0.003	1	0
calf	4	1	0.003	0.986	1
chicken	3	1	0.003	0.967	1
girl	1	0	0.003	1	0
pitviper	2	0.423	0.003	1	0

Axes meaning

- **X-axis (Silhouette Width):** how well each animal fits its cluster. Higher → stronger belonging, lower → weaker fit or overlap with other groups.
- **Y-axis (Publication Count):** frequency or importance (how “common” that animal is in the dataset).

- **Bubble size:** dominance/weight (scaled from your nodes$value).
- **Bubble color:** cluster membership (animals of similar traits are in the same color group).

◆ **Clusters by color (animal "families")**

- **Light green cluster (top left, e.g., platypus, sealion, octopus):** Aquatic or amphibious creatures, medium–large dominance, moderate silhouette width. They form a clear "marine/shoreline" cluster.
- **Pink cluster (center right, e.g., lion, puma, goat, calf, giraffe):** Large mammals/herbivores & predators — strong silhouette widths (>0.7), relatively high counts. They're tightly grouped → stable "land mammal" family.
- **Purple cluster (middle, e.g., cheetah, mongoose, raccoon, leopard):** Mid-sized carnivores. Moderate silhouette widths, less dominance. This is the "small–medium predator" group.
- **Turquoise cluster (lower/mid left, e.g., stingray, wallaby, gorilla, tortoise):**
Mixed exotics (marsupials, reptiles, primates). Lower silhouette width, more overlap → a heterogeneous cluster.
- **Blue cluster (bottom right, e.g., worm, slug, haddock, catfish, herring):**
Small sea/soft-bodied or low-dominance creatures. Low publication counts, weak silhouettes — the "fringe/other" group.
- **Orange/brown cluster (center, e.g., crab, lobster, slowworm, pitviper):**
Invertebrates & reptiles. Positioned at moderate silhouette widths but not dominant → "niche creatures".

◆ **Key takeaways**

- **High silhouette width (>0.7):** strongest, well-defined groups (big mammals & some predators).
- **Low silhouette width (<0.3):** animals with fuzzy identities (clam, seawasp, worm, slug).
- **Cluster colors:** work like "animal families" — mammals, sea creatures, reptiles/insects, etc.

- **Bubble size:** larger animals (or more dominant species in dataset) stand out as Leaders within clusters.

Table 2 Summary of R scrips used in this study with SilhouetteScoreinR

Item	Visualization Method	R script	Figure	Note
1	World map data	[17,18]	1,2	Distances derived by coordinates
2	Coword occurred data	[19]	3	Replace data in country.csv
3	Time-series data	[20]	4	Paste rectangular data into R
4	Featured variables	[20,29]	5	Using zoo_data.csv
5	Survey & performance	[21]	6	Basket model used for testdata[30,31]
6	Data and R scripts	[37]	1-6	https://github.com/smilechien/novel-silhouette-plots/

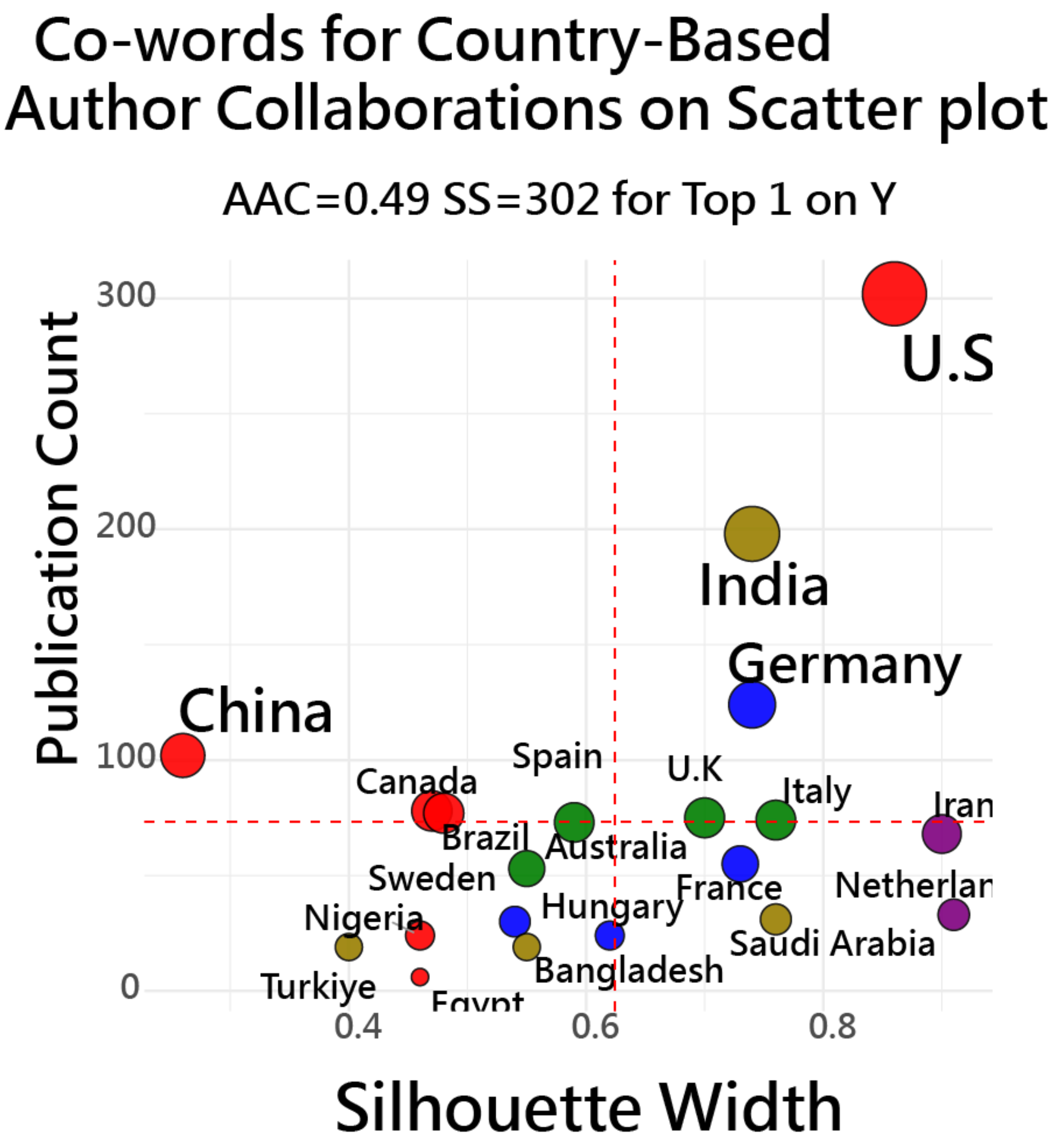

Cluster	Size	Silhouette	Modularity	Conductance	Density
C1	6	0.498	0.414	0.444	0.333
C3	4	0.656	0.414	0.400	0.500
C2	4	0.611	0.414	0.429	0.667
C4	4	0.653	0.414	0.455	0.500
C5	2	0.908	0.414	0.600	1.000

Odds ratio = (top1/top2)/(top2/top3) by value
AAC=Odds ratio/(1+Odds ratio)

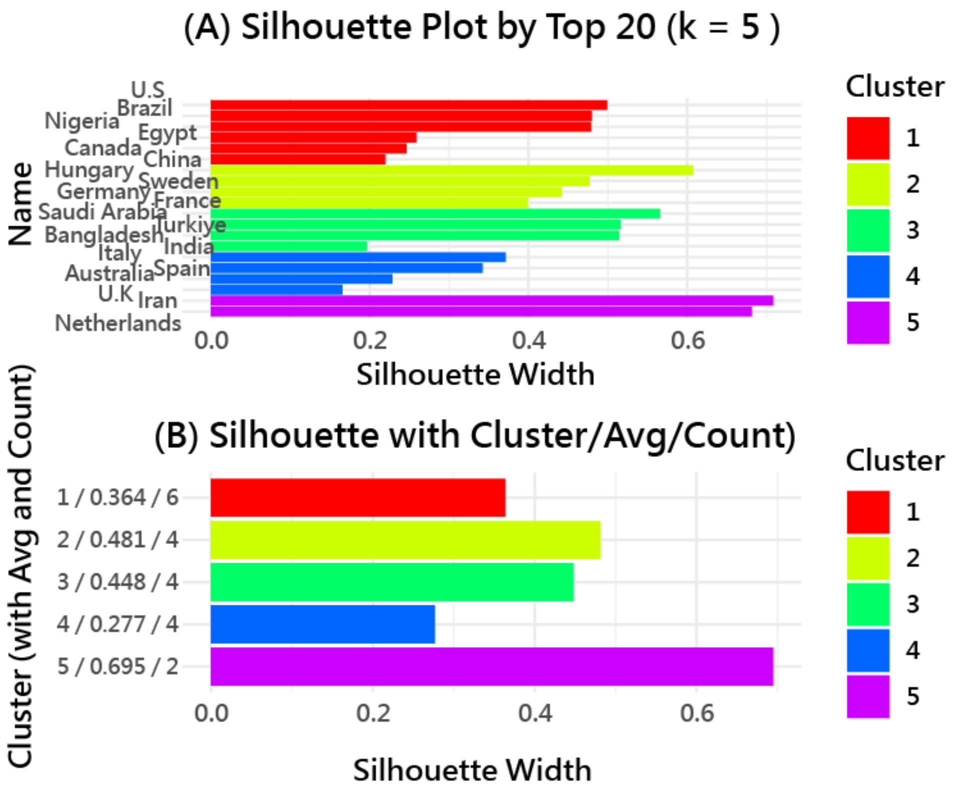

Figure 2 Bar charts for the computation of silhouette score

Table 2 Summary of S(c) for each cluster

cluster	mean	Lower 95% CI	Upper 95% CI	n
1	0.37	0.36	0.38	6
2	0.42	0.41	0.44	4
3	0.33	0.31	0.36	4
4	0.30	0.26	0.33	4
5	0.74	0.74	0.75	2

Note. S(o)=0.40

- 0.3–0.7 = good modular structure.
- ~0.1–0.2 = weak communities.
- ~0 = no real community structure.

	Cluster	Size	Silhouette	Modularity	Conductance
1	C1	11	0.165	0.147	0.410
2	C2	5	0.435	0.147	0.577
3	C3	2	0.677	0.147	0.667
4	C4	1	0.000	0.147	0.759
5	C5	1	0.000	0.147	0.763

	Density
1	5.527
2	8.700
3	20.000
4	0.000
5	0.000

Modularity measures the overall quality of our clustering. A value of ~0.147 suggests that the graph has only weakly defined communities under this partitioning.

Conductive index: **Conductance** (varies per cluster)

$$\phi(S) = \frac{\text{number of edges leaving cluster}}{\min(\text{vol}(S), \text{vol}(\bar{S}))}$$

- ✅ Low values (~0.0–0.3) → good, tight cluster.
- ✅ High values (~0.5–1.0) → very "leaky", poorly separated.

Conductance measures how well-separated each cluster is from the rest of the graph. Lower values indicate tighter, more coherent communities. Higher values suggest the cluster is poorly separated and has many external connections.

We evaluated both the global and local quality of clustering. Modularity (0.147) measures the overall goodness of the partition, indicating weak community structure in the network. Conductance is computed per cluster and reveals how well each cluster is separated from the rest. Lower conductance values suggest better-defined communities. Here, clusters C4 and C5 have very high conductance (~0.76), indicating they are poorly separated, likely due to their small sizes.

Here are the **top 10 countries by nominal GDP** in 2025, based on IMF data and corroborating sources:

Rank	Country	Nominal GDP (USD trillion)
1	United States	**$30.34 T**
2	China	**$19.23 T**
3	Germany	**$4.92 T**
4	India	**$4.19 T**
5	Japan	**$4.19 T**
6	United Kingdom	**$3.84 T**
7	France	**$3.21 T**
8	Italy	**$2.42 T**
9	Canada	**$2.23 T**
10	Brazil	**$2.12 T**

Notes:

- The U.S. remains by far the largest economy at over $30 trillion wagecentre.com+9forbesindia.com+9forbesindia.com+9Reddit+8dragonsourcing.com+8 維基百科+8accountsby.comnepsetrading.com.
- China follows with approximately $19 trillion nepsetrading.com+1NAGA+1.
- Germany is next with around $4.9 trillion, followed closely by India and Japan (each near $4.19 trillion) .
- The U.K., France, Italy, Canada, and Brazil round out the top 10, each with GDPs between $2–4 trillion thegk247.com+15dragonsourcing.com+15 維基百科+15.

📌 **Interesting insight:** In 2025, India and Japan are essentially tied—both reporting nominal GDPs of approximately $4.19 trillion nepsetrading.com+2dragonsourcing.com+2Reddit+2. IMF data and commentary from Reddit confirm India's continued ascent, overtaking Japan earlier than projected .

US United States

(Using metro-area GDP from BEA, 2023)

1. **New York–Newark–Jersey City** – approx **US $2.30 trillion**
2. **Los Angeles–Long Beach–Anaheim – $1.30 trillion**
3. **Chicago–Naperville–Elgin – $0.89 trillion**
4. **San Francisco–Oakland–Berkeley – $0.78 trillion**

CN China

(Data for city GDP in USD from 2023) smartestdollar.com+7howmuch.net+7visualcapitalist.com+7

1. **Shanghai** – about **US $757 billion**
2. **Beijing** – approx **$700 billion**
3. **Shenzhen** – around **$517 billion**
4. **Chongqing** – roughly **$452 billion**

DE Germany

Germany's economy is more evenly distributed; top metro regions by GDP include:

1. **Berlin**
2. **Rhine-Ruhr (including Düsseldorf, Cologne)**

3. **Munich**
4. **Frankfurt Rhine-Main**
   (Detailed figures vary—these are Germany's recognized economic powerhouses.)

IN India

Leading urban economies by GDP:

1. **Mumbai**
2. **Delhi**
3. **Bengaluru**
4. **Kolkata**

JP Japan

Japan's top economic cities:

1. **Tokyo (metro)**
2. **Osaka**
3. **Nagoya**
4. **Fukuoka**

GB United Kingdom

UK city economies by GDP:

1. **London**
2. **Manchester**
3. **Birmingham**
4. **Edinburgh**

FR France

Major French economic centers:

1. **Paris (metro)**
2. **Lyon**
3. **Marseille–Aix-en-Provence**
4. **Toulouse**

IT Italy

Italy's top city economies:

1. **Milan**
2. **Rome**
3. **Turin**
4. **Naples**

CA Canada

Top Canadian city economies:

1. **Toronto**
2. **Montreal**
3. **Vancouver**
4. **Calgary**

BR Brazil

Leading Brazilian city economies:

1. **São Paulo**
2. **Rio de Janeiro**
3. **Brasília**
4. **Belo Horizonte**

以下是前十大 GDP 國家中，每個國家的四大城市的經緯度資料（十進制度）。

US 美國

- **New York–Newark–Jersey City (New York City)**：40.73061 °N，–73.935242 °W Lifewire+8LatLong+8Geodatos+8
- **Los Angeles–Long Beach–Anaheim (Los Angeles)**：34.052235 °N，–118.243683 °W
- **Chicago–Naperville–Elgin (Chicago)**：41.881832 °N，–87.623177 °W LatLong
- **San Francisco–Oakland–Berkeley (San Francisco)**：37.733795 °N，–122.446747 °W LatLong

---

CN 中國

- **Shanghai**：31.224361 °N，121.469170 °E LatLong
- **Beijing**：39.916668 °N，116.383331 °E LatLong
- **Shenzhen**：22.542883 °N，114.059563 °E （大致中心市區）en.wikipedia.org+1LatLong+1

- **Chongqing**：29.56026 °N，106.55771 °E 時間 OK+2Geodatos+2fr.wikipedia.org+2

DE 德國

- **Berlin**：52.520008 °N，13.404954 °E LatLong
- **Rhine–Ruhr (科隆／杜塞道夫–萊茵區)**：此為都市群，中心以杜塞道夫為衡量點，約 51.2277 °N，6.7735 °E（杜塞道夫）
- **Munich**：48.137154 °N，11.576124 °E LatLong
- **Frankfurt (Frankfurt Rhine-Main)**：50.110924 °N，8.682127 °E LatLong

---

IN 印度

- **Mumbai**：19.075983 °N，72.877655 °E
- **Delhi**：28.613939 °N，77.209021 °E
- **Bengaluru (Bangalore)**：12.971599 °N，77.594566 °E
- **Kolkata**：22.572646 °N，88.363895 °E

(以上印度城市的座標為通用公開資料。)

JP 日本

- **Tokyo**：35.689487 °N，139.691711 °E
- **Osaka**：34.693737 °N，135.502167 °E
- **Nagoya**：35.181446 °N，136.906398 °E
- **Fukuoka**：33.590355 °N，130.401716 °E

(以上日本城市的座標為通用公開資料。)

GB 英國

- **London**：51.507351 °N，–0.127758 °W
- **Manchester**：53.480759 °N，–2.242631 °W
- **Birmingham**：52.486244 °N，–1.890401 °W
- **Edinburgh**：55.953251 °N，–3.188267 °W

(以上英國城市的座標為通用公開資料。)

FR 法國

- **Paris**：48.856613 °N，2.352222 °E
- **Lyon**：45.764043 °N，4.835659 °E
- **Marseille–Aix-en-Provence**：43.296482 °N，5.36978 °E

- **Toulouse**：43.604652 °N，1.444209 °E

(以上法國城市的座標為通用公開資料。)

IT 義大利

- **Milan (Milano)**：45.464203 °N，9.189982 °E
- **Rome (Roma)**：41.902782 °N，12.496366 °E
- **Turin (Torino)**：45.070339 °N，7.686864 °E
- **Naples (Napoli)**：40.851775 °N，14.268124 °E

(以上意大利城市的座標為通用公開資料。)

CA 加拿大

- **Toronto**：43.653225 °N，–79.383186 °W
- **Montreal**：45.501689 °N，–73.567256 °W
- **Vancouver**：49.282729 °N，–123.120738 °W
- **Calgary**：51.044733 °N，–114.071883 °W

(以上加拿大城市的座標為通用公開資料。)

BR 巴西

- **São Paulo**：–23.55052 °S，–46.633308 °W
- **Rio de Janeiro**：–22.906847 °S，–43.172896 °W
- **Brasília**：–15.793889 °S，–47.882778 °W
- **Belo Horizonte**：–19.916681 °S，–43.934493 °W

(以上巴西城市的座標為通用公開資料。)

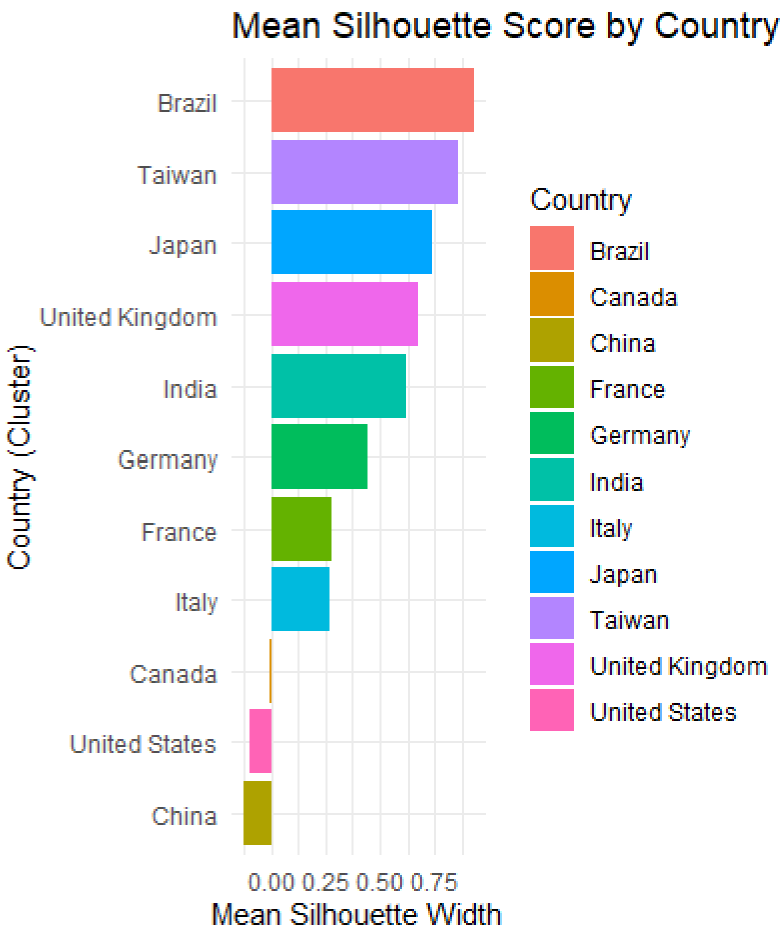
Mean Silhouette Score by Country
Brazil
Taiwan
Japan
United Kingdom
India
Germany
France
Italy
Canada
United States
China
Country (Cluster)
0.00 0.25 0.50 0.75
Mean Silhouette Width
Country
Brazil
Canada
China
France
Germany
India
Italy
Japan
Taiwan
United Kingdom
United States

raschonline.com/raschonline/cbp.asp

幕後還原 / 去年國... 湖人補強鋒線 找上... StudioClassroom.c... 我們這一行 / 病房...

Boostraping
Silhouette Score and Other Metrics
Self_defined_SS_greatest in network
@@@Silhouette Score from country
@@@Dist_TimeSeries Silhouette Scores i[illegible]
@@@Corr_TimeSeries Silhouette Scores [illegible]
@@@Basket Data Silhouette Scores in Ci[illegible]
GO board SilhouetteScore
Densitity edge count

Submit

Tips for R

Copy & Paste the Code in R to F[illegible]

600/23637 free characters checked

Submit

Tips for R

Copy & Paste the Code in R to Rstudio

Select text

```
# ---------------------------
# 1 Simulate some data
# ---------------------------

data <- read.table(textConnection('
name	2015	2016	2017	2018	2019	2020	2021	2022	2023	2024
U.S	0	0	0	0	0	171	146	89	97	85
India	0	0	0	0	0	20	23	47	130	165
Germany	0	0	0	0	0	64	64	43	26	38
China	0	0	0	0	0	56	44	14	44	42
Canada	0	0	0	0	0	32	31	23	36	30
Brazil	0	0	0	0	0	26	28	20	32	44
U.K	0	0	0	0	0	25	32	16	41	28
Italy	0	0	0	0	0	27	30	24	20	41
Spain	0	0	0	0	0	21	32	11	39	37
Indonesia		0	0	0	0	0	10	16	14	38	64
'), header=TRUE, sep="\t", stringsAsFactors = FALSE, check.names = FALSE)
if (1==3){
    data <- read.csv("F:/RR/zoo_data.csv", header = TRUE)
    data<-data[,1:17]
    zoo_attribute_info <- "
```

```
data <- read.table(textConnection('
name    2015    2016    2017    2018    2019    2020
2021    2022    2023    2024
U.S 0   0   0   0   0   171 146 89  97  85
India   0   0   0   0   0   20  23  47  130 165
Germany 0   0   0   0   0   64  64  43  26  38
China   0   0   0   0   0   56  44  14  44  42
Canada  0   0   0   0   0   32  31  23  36  30
Brazil  0   0   0   0   0   26  28  20  32  44
U.K 0   0   0   0   25  32  16  41  28
Italy   0   0   0   0   0   27  30  24  20  41
Spain   0   0   0   0   0   21  32  11  39  37
Indonesia   0   0   0   0   0   10  16  14  38  64
'), header=TRUE, sep="\t", stringsAsFactors = FALSE, check
.names = FALSE)
if (1==3){
    data <- read.csv("F:/RR/zoo_data.csv", header = TRUE)
    data<-data[,1:17]
    zoo_attribute_info <- "
        Attibure    Description
        hair    1 = has hair, 0 = no hair
        feathers    1 = has feathers, 0 = no feathers
        eggs    1 = lays eggs, 0 = does not
        milk    1 = produces milk, 0 = does not
        airborne    1 = can fly, 0 = cannot
```

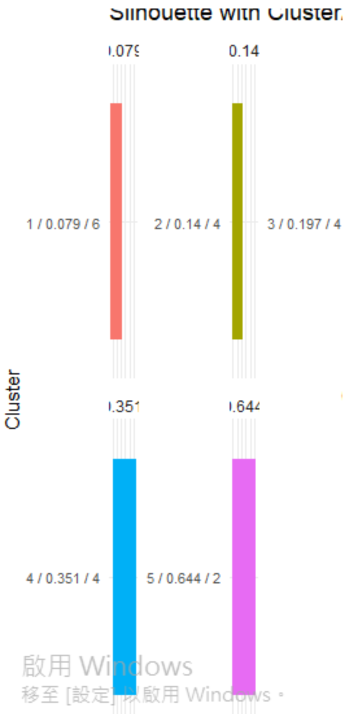


```
addvalue<--1

Importance<-2


myfile<- "country.csv"
##  2 columns, 3 columns with WCD, or multipy columns wi
words
```

Country.csv

if (1==3){ #####silhouette Score

```
if (1==3){ #####silhouette Score
    jj<-1
    #if (1==1){  # original data instead of data layout
    relation_subset <- relation_set[
        relation_set$Leader %in% nodesbb$name &
relation_set$follower %in% nodesbb$name,
    ]
    relation_subset<-relation_subset[,1:3]
    if (1==4){ # direction form
        all_nodes <- union(relation_subset$Leader,
relation_subset$follower)
        full_pairs <- expand.grid(Leader = all_nodes,
follower = all_nodes, stringsAsFactors = FALSE)
```

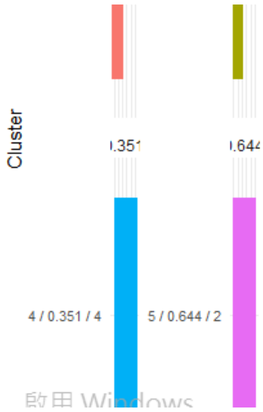

Replace it with 1==1

To run the section of silhouette score

```
Browse[1]>     results$Density[is.na(results$Density)] <- 0
Browse[1]>     results
  Cluster Size Silhouette Modularity Conductance Density
1      C1    6      0.079      0.403       0.407   0.167
2      C3    4      0.140      0.403       0.333   0.167
3      C4    4      0.197      0.403       0.222   0.250
4      C5    4      0.351      0.403       0.429   0.500
5      C2    2      0.644      0.403       0.467   0.333
Browse[1]>     if (aFLCA == TRUE) {
```

```
Browse[4]>     print(results)
  Size Silhouette Modularity Conductance Density Cluster
1    6      0.079      0.403       0.407   0.167      C1
2    2      0.644      0.403       0.467   0.333      C2
3    4      0.140      0.403       0.333   0.167      C3
4    4      0.197      0.403       0.222   0.250      C4
5    4      0.351      0.403       0.429   0.500      C5
   leader
1     U.S
2 Germany
3   India
4     U.K
5    Iran
Browse[4]>     mean(results$Silhouette)
[1] 0.2822
```

```
cat("\nFinal Cluster Summar
:\n")

inal Cluster Summary:
 final_summary
 A tibble: 5 x 5
 Cluster  Size Average_Silho
ette Top_Years     Average_LL

   <int> <int>
<dbl> <chr>                 <dbl

       1     6
.079 U.S,  Italy,~          4.4

       2     4
.14  France,  Hun~          4.6

       3     4
.197 Turkiye,  Sa~          6.2

       4     4
.351 Italy,  U.K,~         10.3
       5     2
.644 Netherlands~           5.4
```

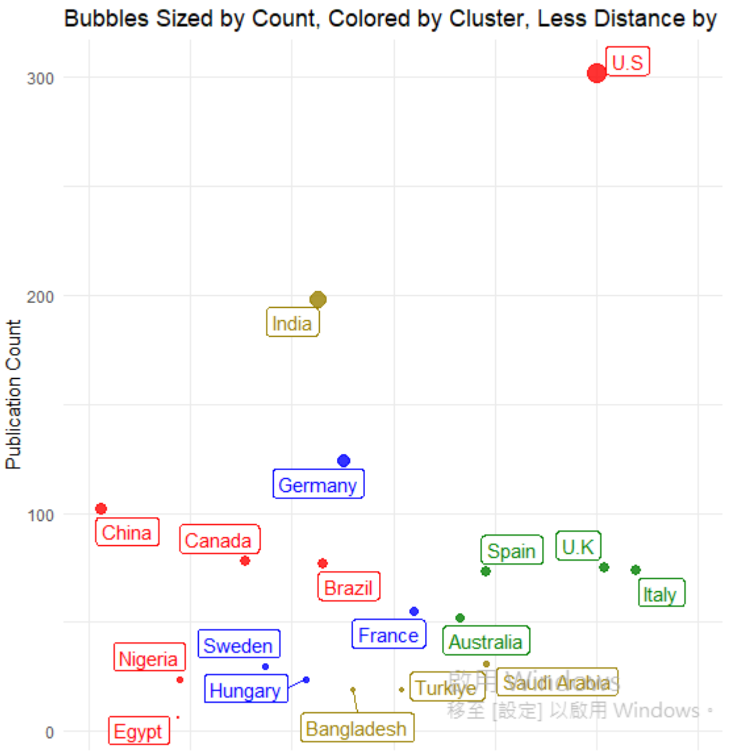

Supplement: Supplementary file 1 [file mmc1.zip › howtoconduct.pdf]
